# Parental Depression Predicts Child Body Mass via Parental Support Provision, Child Support Receipt, and Child Physical Activity: Findings From Parent/Caregiver–Child Dyads

**DOI:** 10.3389/fpsyg.2020.00161

**Published:** 2020-02-07

**Authors:** Karolina Zarychta, Anna Banik, Ewa Kulis, Monika Boberska, Theda Radtke, Carina K. Y. Chan, Aleksandra Luszczynska

**Affiliations:** ^1^Faculty of Psychology, SWPS University of Social Sciences and Humanities, Wrocław, Poland; ^2^Department of Psychology and Psychotherapy, Witten/Herdecke University, Witten, Germany; ^3^School of Psychology and Public Health, College of Science, Health and Engineering, La Trobe University, Melbourne, VIC, Australia; ^4^Trauma, Health, and Hazards Center, University of Colorado at Colorado Springs, Colorado Springs, CO, United States

**Keywords:** depression, social support, physical activity, body mass, dyads, parent, child

## Abstract

**Objective:**

Although there is substantial evidence corroborating the within-individual associations between depression, social support, moderate-to-vigorous physical activity (MVPA), and body mass, much less is known about across-individual associations. This study investigated the indirect associations between parental depression and objectively measured body mass in children. In particular, it was hypothesized that higher levels of parental depression (measured at Time 1, T1) would explain higher levels of child body mass in children (assessed at Time 2, T2), via three mediators, namely parental reports of provision of MVPA support (T1), child reports of receipt of MVPA support (T1), and child MVPA (T2).

**Design:**

Parent–child dyads provided self-reports twice, at baseline (T1) and 7- to 8-month follow-up (T2). A total of 879 dyads were enrolled (1,758 individuals; 5- to 11-year-old children, 52.4% girls, 83.2% mothers). Body weight and height were measured objectively. Manifest path analyses were performed to test the indirect effects.

**Results:**

Analyses corroborated the assumed indirect effects: high levels of depression in parents (T1) were indirectly associated with high levels of body mass in children (T2), via three mediators: low levels of parental support provision (T1), low levels of child support receipt (T1), and low levels of child MVPA (T2). The alternative models assuming that either parental support provision or child support receipt can be excluded as the mediators yelded a poor model-data fit. The hypothesized mediation effects were corroborated when controlling for the baseline levels of parental and child MVPA and body mass.

**Conclusion:**

The findings confirm complex across-individual effects of parental depression on high levels of body mass in children. Parental mental health may contribute to the childhood obesity epidemic.

## Introduction

The prevalence of youth overweight and obesity increased from 4% in 1975 to over 18% in 2016, with over 340 million 5- to 19-year-old children and adolescents being overweight or obese (World Health Organization [WHO], 2018a). To prevent such an unfavorable increase in child body mass, World Health Organization [WHO], 2018b) recommends performing at least 60 min of moderate-to-vigorous physical activity (MVPA) daily. Regular physical activity (PA) is not only a well-recognized protective factor for overweight and obesity prevention, but also contributes to the prevention of many non-communicable diseases, e.g., stroke, diabetes, or colon cancer (Stanaway et al., 2018). Yet, the prevalence of children meeting recommended PA levels remains low (World Health Organization [WHO], 2018b).

Social–ecological models of childhood obesity (Davison and Birch, 2001) indicate a range of child and family characteristics (e.g., parental and child MVPA) among the crucial determinants of excessive body mass in children. Individual and environmental factors (e.g., social support provision and receipt) have been reported to explain child PA (Sallis et al., 2006). For example, the association between parental support for PA and child PA was confirmed in the review of 19 studies (including 16 cross-sectional ones) (Gustafson and Rhodes, 2006). Similarly, a systematic review of 96 research confirmed parental effects (such as support for PA provision) on child PA (Edwardson and Gorely, 2010). Yet, the effects were consistent only cross-sectionally, while 11 longitudinal studies provided mixed findings (Edwardson and Gorely, 2010). Also, parental support variables were moderately associated with child PA in a meta-analysis of 94 cross-sectional and 18 longitudinal studies (Yao and Rhodes, 2015). In sum, theoretical and empirical links between parental support variables, child PA, and child body mass are well-established, yet the evidence is limited due to the cross-sectional design of the majority of previous studies. Moreover, as emphasized in social–ecological models (Davison and Birch, 2001; Sallis et al., 2006), child behaviors (i.e., MVPA) may be the direct and proximal predictors, while the social factors might be the indirect, more distant predictors of childhood obesity. Therefore, testing indirect associations between social support, PA, and body mass is needed. Additionally, as the majority of research accounted for self-reports of children or parents only, but not both (see Yao and Rhodes, 2015), the effects of perceptions of parents and children (e.g., social support provision reported by parents and social support receipt reported by children) should be investigated jointly. Last but not least, child’s age may play a role. Systematic reviews indicated that the effects of parental behaviors and cognitions on child PA and obesity are significant among 5- to 11-year-old children. In contrast, the effects of parental behaviors and cognitions on behaviors of adolescents (aged ≤ 12 years old) may be small or non-significant, due to the increasing influence of peers’ cognitions and behaviors (Cislak et al., 2012). Thus, research investigating parental determinants of child PA and body mass should account for the specific age group, namely 5- to 11-year-old children.

The global action plan for PA (World Health Organization [WHO], 2018b) suggests that parents are facing a difficult task of providing support for child PA to obtain or maintain optimal body mass of their children. Therefore, thorough research is needed to identify the factors that may affect parental ability to support child PA (Masarik and Conger, 2017).

The family stress model (FSM; Masarik and Conger, 2017) explains child health and wellbeing by parental distress and by the consequences of parental distress. In particular, the FSM suggests that parental psychological distress leads to disrupted parenting, which results in child adjustment problems. FSM may be applied regardless of the evaluation of the type and presence of the stressors preceding distress; the focus is rather on the mental health issues resulting from distress and the links between mental health issues, parental behaviors, and child health/adjustment outcomes (Masarik and Conger, 2017). For example, the FSM suggests that parental depression is leading to unfavorable parenting practices. It was found that parental depression is prospectively associated with unsupportive parenting practices (Newland et al., 2013), a low level of provision of social support (Nievar et al., 2014), less time spent with children (Iruka et al., 2012), and punitive behaviors toward children (Emmen et al., 2013). In turn, these parental behaviors are prospectively linked to unhealthy behaviors and excessive body weight in children (McCurdy et al., 2010).

The associations between parental depression, social support provision, child PA, and child body mass were already investigated (Wilson and Durbin, 2010). However, the majority of research has used cross-sectional design, or accounted for only two or three variables from the depression – support – MVPA – body mass chain, or focused on bivariate associations instead of complex, indirect effects. For example, a meta-analysis of 28 studies (Wilson and Durbin, 2010) indicated that parental depression had small-to-moderate effects on parental disengagement and parental support behaviors (e.g., withdrawal, showing less affection, lower provision of support). These effects occurred among mothers and fathers. Yet, all analyzed studies relied on parental reports of support provision only. Furthermore, children who were exposed to maternal depressive symptoms as toddlers were more likely to have low MVPA levels at the age of 4 to 6 years old, compared to children who did not experience maternal depression (Fernald et al., 2008). Again, the study was based on parental reports only. The findings of a dyadic cross-sectional study (Schoeppe and Trost, 2015) showed positive associations between maternal and paternal support for PA with PA of preschool-aged children. Yet another cross-sectional study relying on dyadic data collection (*N* = 4,601 dyads) showed that parental depression was related to low levels of parenting quality (defined as a combination of parental perceptions of family cohesion and child support receipt), low levels of child PA, and high levels of child body mass (McConley et al., 2010). Unfortunately, the cross-sectional character of data limits any conclusions regarding the order in which these variables operate. Additionally, the parenting quality variable was calculated as a combination of parental reports of cohesion and child reports of support receipt; therefore, it is impossible to disentangle the effects of child perceptions and parental perceptions of social support (McConley et al., 2010).

This prospective study tested indirect across-individual associations between parental depression and child body mass, in the dyadic context of parents’ caregiving for their 5- to 11-year-old children. In particular, it was hypothesized that higher levels of parental depression symptoms [measured at Time 1 (T1)] would predict higher levels of child body mass [measured at Time 2 (T2)] indirectly, via three mediators operating in a sequence: lower levels of parental provision of PA support (T1), lower levels of child receipt of PA support from parents (T1), and lower child MVPA (T2). The assumed indirect effects were investigated controlling for child gender, age, T1 levels of child MVPA, child body mass (T1), parental gender, age, T1 and T2 levels of parental MVPA, and parental body mass (T1).

## Materials and Methods

### Participants

Parent–child dyads were invited to take part in a larger study, exploring determinants of child PA and body mass (see Horodyska et al., 2018; Liszewska et al., 2018; Zarychta et al., 2019).

Parents (99.6%) or legal guardians (0.4%; including adoptive parents) were the main caregivers regarding the time spent with their child and co-organizing child PA. Children with physical impairments leading to major movement disabilities (e.g., cerebral palsy) were excluded. No additional exclusion criteria were applied. Regarding younger children (aged 5–7 years old), only those who attended primary schools and reached cognitive, physical, and social maturity levels required to start the first grade (as evaluated by a professional education counsellor) were included.

At T1 (baseline), 879 dyads (1,758 individuals) participated in the study. Parents (*N* = 879) were women (83.2%) and men (16.8%), aged 24–68 years old (*M* = 36.65, *SD* = 6.10), with a body mass index (BMI) ranging from 16.14 to 41.61 (*M* = 24.43, *SD* = 3.94). The majority of parents (60.1%) had normal body weight, 29.1% were overweight, 8.9% were obese, and 1.9% were underweight. Further, the majority of parents had either higher education (39.8%) or secondary education (28.9%), whereas the remaining parents declared vocational (14.4%), post-secondary (11.9%), or primary (5.0%) education. The distribution of the education levels in the analyzed sample was similar to those found in the general population in Poland (Central Statistical Office, 2015). More than a half of the parents (56.1%) evaluated that their economic status was similar to the economic status of the average family in Poland, the remainder indicated their economic status to be better (32.5%), or worse (11.4%).

Children (*N* = 879) were girls (52.4%) and boys (47.6%), aged 5–11 years old (*M* = 8.46, *SD* = 1.34); 0.7% were 5 years old, 9.8% were 6 years old, and 89.5% were 7- to 11-years-old. Accounting for the International Obesity Task Force (IOTF) cut-off points (Cole and Lobstein, 2012), 67.9% of children had normal body weight, 17.7% were overweight, 7.3% were obese, and 7.1% were underweight. All parent and child participants were Caucasian (as 98% of Poland’s population; Central Statistical Office, 2015).

At T2 (7- to 8-month follow-up), 68.3% of the T1 respondents (603 dyads; 1,206 individuals) agreed to participate. The full information maximum likelihood (FIML) procedure was used to account for data missing due to the longitudinal dropout at T2, thus, data collected from 879 dyads (1,758 individuals) were included in the analyses.

### Procedure

Data were collected twice, at baseline (T1) and at 7- to 8-month follow-up (T2). Potential participants were approached between 2011 and 2015 in schools and general practitioners’ offices in six regions of Poland. To represent economic diversity, data were obtained from locations in the regions characterized as lower in economic development (23% of locations), medium in economic development (50%), and higher in economic development (27%) (based on Poland’s economic development index; Central Statistical Office, 2015). Participants were informed about the aims of the study and the research schedule. Informed consent was collected from parents (about their own and child’s participation) and assent was obtained from children. De-identified codes were assigned to participants to ensure anonymity across the measurement points. Younger children (aged 5- to 8-years-old) were interviewed using a structured interview schedule. Older children (aged 9–11 years old) and parents completed a questionnaire, unless they preferred being interviewed. Parents and children completed the questionnaires separately. All participants were offered a small thank you gift (e.g., a pen, a notebook) at T1 and T2.

At T1, children responded to the questions about support receipt and MVPA, while parents provided data on their own depression symptoms, support provision, MVPA, education, and perceived economic status. Participants’ body weight and height were measured with certified scales and rods. At T2, study personnel revisited the schools, practitioners’ offices or participants’ homes after contacting parents by phone to repeat the measurements. The time gap between the measurement points was chosen because it comprises one school year [from the beginning of the school year (T1) to its end (T2)]. Thus, the dropout due to school change after the completion of a school year was limited. The attrition occurred because (a) either parents or children decided to discontinue their participation or (b) either parents or children were not available at T2.

Before the data collection reported in the present study, a qualitative pilot study with *N* = 18 children (aged 5–11 years old) was conducted to check the comprehension of the items assessing PA. Children were asked to explain the instructions and the items in their own words and to indicate any phrases they do not understand or are unsure of. The pilot study indicated that using provided instructions and the items, children were able to correctly classify their behaviors, referring to light, moderate, and vigorous PA.

The study was approved by the Internal Review Board at the SWPS University of Social Sciences and Humanities, Wrocław, Poland. All procedures were in accordance with the ethical standards of the institutional research ethics committee and in line with the 1964 Helsinki declaration and its later amendments.

### Materials

The measures were administered in Polish. The respective Polish language versions of the measures were applied in research conducted among children and adults (e.g., Koziara, 2016; Zarychta et al., 2018). Means, standard deviations, and reliability coefficients for all measures are presented in Table 1.

**TABLE 1:** Descriptive statistics, reliability, and correlations between the study variables (*N* = 879 parent–child dyads).

		*M (SD)*	α	2	3	4	5	6	7	8	9	10	11	12	13	14
1.	Depression (P, T1)	11.02 (9.03)	0.90	−0.20***	−0.10**	0.03	–0.02	–0.06	−0.09**	0.02	0.03	0.01	–0.09	0.04	0.06	<0.01
2.	Support (P, T1)	15.57 (3.35)	0.84		0.54***	0.13***	0.14***	0.18***	0.24***	–0.02	–0.08	–0.05	0.17***	0.05	–0.01	−0.14***
3.	Support (Ch, T1)	14.41 (3.75)	0.78			0.11**	0.12***	0.30***	0.25***	–0.01	0.02	0.03	0.05	0.05	–0.02	−0.12***
4.	MVPA (P, T1)	21.95 (19.32)	0.53				0.62***	0.20***	0.08	−0.09**	–0.01	–0.02	–0.02	–0.01	–0.03	–0.04
5.	MVPA (P, T2)	22.13 (16.96)	0.59					0.10**	0.17***	–0.03	0.01	<0.01	–0.01	–0.01	–0.04	–0.04
6.	MVPA (Ch, T1)	44.64 (27.36)	0.56						0.34***	<0.01	–0.01	0.01	0.03	0.05	0.02	−0.12**
7.	MVPA (Ch, T2)	46.49 (25.11)	0.53							0.03	0.05	0.02	0.07	0.06	0.01	−0.11**
8.	BMI (P, T1)	24.44 (3.91)									0.17***	0.16***	0.19***	0.11**	−0.29***	–0.02
9.	BMI *z*-score (Ch, T1)	0.44 (1.24)										0.94***	−0.10**	0.08	0.01	–0.05
10.	BMI *z*-score (Ch, T2)	0.30 (1.24)											−0.10**	0.07	0.02	–0.04
11.	Age (P, T1)	36.64 (6.09)												0.19***	−0.19***	<0.01
12.	Age (Ch, T1)	8.46 (1.34)													–0.08	–0.01
13.	Gender (P)															0.03
14.	Gender (Ch)															

***p < 0.001; **p < 0.01; T1 = Time 1, baseline; T2 = Time 2, 7- to 8-month follow-up; P, parent; Ch, child; Support, social support provision (parents) and support receipt (children); MVPA, moderate-to-vigorous physical activity; BMI, body mass index *z*-score (children) and body mass index (parents).

#### Depression Symptoms (T1)

Parental depression was measured with the 20-item Center for Epidemiological Studies – Depression (CES-D) Scale (Radloff, 1977). Parents were asked how often over the past week they experienced symptoms associated with depression, e.g., “I felt that everything I did was an effort.” The responses ranged from 0 (*rarely or none of the time*) to 3 (*most or almost all the time*). Higher total scores represent a higher level of parental depressive symptoms. The average sum score was *M* = 11.02, *SD* = 9.03, α = 0.90.

#### Social Support Provision and Social Support Receipt (T1)

Perceived parental provision of PA support (henceforth: parental support provision) and perceived child receipt of PA support (henceforth: child support receipt) were measured with five items each (based on Edwardson and Gorely, 2010). Parents were asked about different types of PA support (encouragement, transport, attitudes, organization, supervision), e.g., “I take my child to the places where they can play sports.” Children were asked about support they get from their parents, e.g., “My parents take me to the places where I can play sports.” The responses ranged from 1 (*definitely not*) to 4 (*definitely yes*). Higher total scores represent a higher level of parental support provision or child support receipt. The average sum score was *M* = 15.57, *SD* = 3.35, α = 0.84 at T1. For child support receipt, the sum score average level was *M* = 14.41, *SD* = 3.75, α = 0.78 at T1.

#### Moderate-to-Vigorous Physical Activity (T1 and T2)

Parental and child MVPA levels were assessed by the Godin Leisure-Time Exercise Questionnaire (Godin and Shephard, 1985), which was found to have acceptable validity and reliability among adults (Godin and Shephard, 1985) and 7- to 15-year-old children (Koo and Rohan, 1999). Verbal instructions were provided at the beginning of the interview (or filling out the questionnaires) to clarify the differences between light, moderate, and vigorous PA, with a reference to heart beating, sweating, and ability to talk while exercising, followed by examples of light-intensity, moderate-intensity, and vigorous-intensity exercises. Participants were asked to provide an open-ended response about the daily number of any “vigorous (heart beats faster, you are sweating) PA sessions lasting at least 15 min,” and “moderate (not so exhausting) PA sessions lasting at least 15 min” during last week. Examples of MVPA were provided. The MVPA index [accounted for PA bouts (15 min) and the metabolic values of PA per week] was calculated with a formula: MVPA score = 9 × (vigorous bouts per week) + 5 × (moderate bouts per week) (Godin and Shephard, 1985). The average levels of parental MVPA at T1 were *M* = 21.95, *SD* = 19.32, α = 0.53, and *M* = 22.13, *SD* = 16.96, α = 0.59 at T2. The mean levels of child MVPA at T1 were *M* = 44.64, *SD* = 27.36, α = 0.56, and *M* = 46.49, *SD* = 25.11, α = 0.53 at T2. The values of the intraclass correlation coefficient for parental MVPA and child MVPA were 0.23, *p* < 0.001 at T1, and 0.17, *p* < 0.001 at T2.

#### Parental Body Weight and Height (T1) and Child Body Weight and Height (T1 and T2)

Parental and child body weight and height were assessed objectively with standard medically approved telescopic height measuring rods and floor scales (scale type: BF-100 or BF-25; Beurer, Germany, measurement error < 5%). For parents, BMI was calculated using body weight and height: BMI = weight (kg)/height^2^ (m^2^). The average levels of parental BMI were *M* = 24.44, *SD* = 3.91 at T1.

For children, age- and gender-specific BMI *z*-score values were calculated with WHO AnthroPlus macro (World Health Organization [WHO], 2011) and used across analyses. The average levels of child BMI *z*-score were *M* = 0.44, *SD* = 1.24 at T1, and *M* = 0.30, *SD* = 1.24 at T2.

### Data Analysis

The G^∗^Power calculator (Faul et al., 2007) was used to determine the sample size. Assuming small effect sizes of the self-reported variables on objectively measured body mass (*f*^2^ = 0.03) and accounting for potential confounders (listed below), the determined sample size was 900 dyads.

Analyses were performed using SPSS version 24 and IBM AMOS 25. Path analyses with maximum-likelihood estimation were conducted (Byrne, 2010). Little’s MCAR test indicated that the missing data patterns were systematic, Little’s χ^2^(417) = 497.38, *p* = 0.004. To reduce the potential negative impact of a systematic dropout, missing data were accounted for with a FIML estimation procedure, recommended for data with a systematic attrition (Graham, 2009). Mardia’s coefficient of multivariate normality indicated a moderate level of non-normality (35.43 for the hypothesized model).

Several model-data fit indices were applied. A cut-off point of ≤0.08 for the root mean square error of approximation (RMSEA) was used (Byrne, 2010). A cut-off point of ≥0.90 for the comparative fit index (CFI), Tucker–Lewis index (TLI), and the normed fit index (NFI) may be considered acceptable, whereas values ≥0.95 represent good model-data fit (Byrne, 2010). The indirect effects were evaluated with their unstandardized effect coefficients, after applying 10,000 bootstraps (95% confidence intervals).

The hypothesized model assumed that the independent variable, parental depression (T1), would be associated with three mediators [parental support provision (T1), child support receipt (T1), and child MVPA (T2)], that in turn would predict child BMI *z*-score (T2). Ideally, each mediator should be measured at a different time point to establish a temporal precedence (MacKinnon, 2008). As the present study used only two measurement points, we decided to include T1-mediator indicators for social support variables and T2-mediator indicators for MVPA variables (controlling for MVPA at T1). Social support and MVPA behavior constitute two distinct categories of variables (social influence variables and health behaviors). Social influence variables are assumed to be determinants of health behaviors and to precede health behaviors (see theoretical models, e.g., Davison and Birch, 2001; Sallis et al., 2006). These determinants are usually measured at an earlier time point than health behaviors (for a similar approach see e.g., Zarychta et al., 2019).

The first analysis was conducted for the unconstrained hypothesized model. In case the indirect effect of the parental depression on child BMI *z*-score would occur in this model, it may be statistically significant, even if one of the component paths is not significant. That is, a significant indirect effect could occur in the unconstrained hypothetical model, even if the effects of parental depression on either parental support provision or child support receipt would be non-significant.

In the next step, a series of nested models assuming alternative indirect effects, was tested. The first nested model assumed that the chain of variables would be significant: parental depression (T1) → parental support provision (T1) → child MVPA (T2) → child BMI *z*-score (T2). All other pathways (from and to other mediators in the model) were constrained to zero. The second nested model assumed that the second chain of variables would be significant: parental depression (T1) → child support receipt (T1) → child MVPA (T2) → child BMI *z*-score (T2). Again, all other pathways (from and to other mediators in the model, e.g., from parental depression to parental support provision) were constrained to zero. The third nested model assumed the simple mediation [parental depression (T1) → child MVPA (T2) → child BMI *z*-score (T2)], with remaining pathways (from and to other mediators in the model, e.g., from parental depression to parental support provision) constrained to zero. Finally, the fourth nested model assumed that in line with the hypothesis, there would be three mediators operating in a sequence: parental depression (T1) → parental support provision (T1) → child support receipt (T1) → child MVPA (T2) → child BMI *z*-score (T2). Again, the remaining pathways (e.g., between parental depression and child support receipt) were constrained to zero.

The following covariates were accounted for: child MVPA at T1, child BMI *z*-score at T1, parental and child age (T1), parental and child gender, parental MVPA (T1 and T2), and parental body mass (T1). All parental and child T1 variables were assumed to covary.

Finally, sensitivity analyses were conducted to test if the pattern of the associations is similar in the hypothesized model, compared to the model which controlled for child MVPA (T1) and child BMI *z*-score (T1) only.

## Results

### Preliminary Analyses

Parents who completed T1 and T2 measurements did not differ from dropouts in terms of depression, parental support provision, MVPA, BMI, age, all *F*s < 2.31, *p*s > 0.129, or gender, χ^2^(1) = 0.94, *p* = 0.332. Children who participated at both T1 and T2 measurements did not differ from dropouts in terms of child support receipt, MVPA, BMI *z*-score, all *F*s < 2.25, *p*s > 0.134, or gender, χ^2^(1) = 0.69, *p* = 0.405. Child dropouts and child completers differed in terms of age, *F*(1,878) = 19.46, *p* < 0.001, with child dropouts being older (*M* = 8.52, *SD* = 1.51) than completers [*M* = 8.44, *SD* = 1.26, Cohen’s *d* = 0.06 (95% CI: −0.03, 0.15)]. Child BMI *z*-score significantly decreased from T1 to T2, *t*(1,878) = 9.29, *p* < 0.001. Bivariate correlations between study variables (for the total sample of *N* = 879 dyads) are presented in Table 1.

### Findings for the Hypothesized Model

**FIGURE 1:**
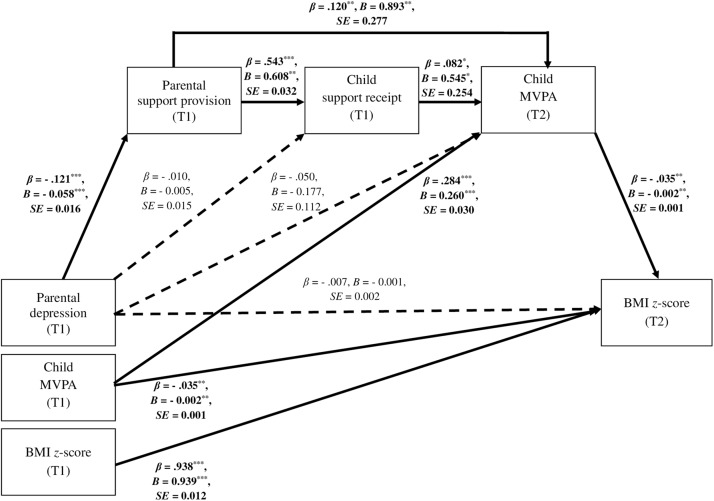
Direct associations between parental depression, child MVPA, child BMI *z*-score, parental support provision, and child support receipt (*N* = 879 parent–child dyads) in the hypothesized unconstrained model. ^∗∗∗^*p* ≤ 0.001; ^∗∗^*p* < 0.01; ^∗^*p* < 0.05; T1 = Time 1, baseline; T2 = Time 2, 7- to 8-month follow-up. Only significant and unstandardized effect coefficients (*B*) are presented along bold arrows. All parental and child predictors at T1 and control variables (T1) were assumed to covary. Residuals of the mediators (T1, T2) were assumed to covary. For clarity, all associations between covariates are not displayed. The covariates include: child MVPA at T1, child BMI *z*-score at T1, parental and child age (T1), parental and child gender, parental MVPA (T1 and T2), and parental BMI (T1). For values of all path, correlation, and covariance coefficients see Supplementary Table 1.

The unconstrained hypothesized model, calculated for *N* = 879 dyads, had a good fit, with χ^2^(27) = 95.53, *p* < 0.001, χ^2^/df = 3.54, NFI = 0.971, TLI = 0.927, CFI = 0.978, RMSEA = 0.054 (90% CI: 0.042, 0.066) (Table 2). Direct associations between the independent variable (T1), the three mediators (T1 and T2, respectively), and the dependent variable (T2) are presented in Figure 1 and Supplementary Table 1. The variables in the model explained 14% of child MVPA (T2) and 88% of child body mass (T2).

**TABLE 2:** The fit coefficients for the tested models [including the hypothesized unconstrained model and the nested (constrained) models], and indirect effects between parental depression and child BMI *z*-score (*N* = 879 parent–child dyads) for well-fitted models.

The model: assumed indirect pathways	Model-data fit indices						Indirect effect^b^		
	χ^2^(df)^a^	χ^2^/df	NFI	TLI	CFI	RMSEA (90% CI)	Unstandardized estimate	SE	99% CI
**The hypothesized unconstrained models**									
The hypothesized unconstrained model with covariates:									
depression (P, T1) → the mediators [support provision (P, T1), support receipt (Ch, T1), MVPA (Ch, T2)] → BMI *z*-score (Ch, T2)	**95.85 (27)**	**3.54**	**0.970**	**0.927**	**0.978**	**0.054 (0.042, 0.066)**	**0.0004**	**0.0002**	**<0.0001, 0.0014**
The hypothesized unconstrained model without covariates (except for child MVPA and BMI *z*-score at T1):									
depression (P, T1) → the mediators [support provision (P, T1), child support receipt (Ch, T1), MVPA (Ch, T2)] → BMI *z*-score (Ch, T2)	**11.67 (3)**	**3.20**	**0.995**	**0.975**	**0.996**	**0.050 (0.021, 0.082)**	**0.0004**	**0.0002**	**<0.0001, 0.0014**
**The constrained nested models^c^**									
First nested model:									
depression (P, T1) → support provision (P, T1) → MVPA (Ch, T2) → BMI *z*-score (Ch, T2)	410.93 (31)	13.26	0.874	0.648	0.880	0.118 (0.108, 0.129)	Does not apply		
Second nested model:									
depression (P, T1) → support receipt (Ch, T1) → MVPA (Ch, T2) → BMI *z*-score (Ch, T2)	425.90 (31)	13.74	0.870	0.635	0.876	0.121 (0.110, 0.131)	Does not apply		
Third nested model:									
depression (P, T1) → MVPA (Ch, T2) → BMI *z*-score (Ch, T2)	446.55 (32)	13.95	0.863	0.628	0.869	0.122 (0.112, 0.132)	Does not apply		
Fourth nested model:									
depression (P, T1) → support provision (P, T1) → support receipt (Ch, T1) → MVPA (Ch, T2) → BMI *z*-score (Ch, T2)	**109.43 (30)**	**3.65**	**0.966**	**0.924**	**0.975**	**0.055 (0.044, 0.066)**	**0.0001**	**<0.0001**	**<0.0001, 0.0002**

*^*a*^All χ^2^-values were significant (*p* < 0.001), except for the unconstrained hypothesized model without covariates (*p* = 0.009). ^*b*^The indirect effects were calculated only for models that had acceptable fit. ^*c*^The nested models assumed that besides the indicated indirect path, all remaining paths (to and from the mediators) that might contribute to the estimation of a respective indirect effect (of the respective independent variable on the respective outcome variable) are constrained to zero. CI, confidence intervals (bootstrap-based, 10,000 repetitions). Significant values of indirect effect coefficient are marked in bold. Fit indices indicating acceptable model-data fit are marked in bold. T1 = Time 1, baseline; T2 = Time 2, 7- to 8-month follow-up; P, parent; Ch, child; MVPA, moderate-to-vigorous physical activity.*

The analysis of the unconstrained hypothesized model (Table 2) showed a significant indirect effect of parental depression (T1) on child body mass (T2), via the three mediators, namely parental support provision (T1), child support receipt (T1), and child MVPA (T2), the unstandardized estimate = 0.0004, *SE* = 0.0002, 99% CI (<0.0001, 0.0014). Regarding the direct effects, the majority were significant (Figure 1 and Supplementary Table 1). However, three direct effects included in the model were not significant. For example, there was no significant direct association between parental depression (T1) and child support receipt (T1), *p* = 0.723; similarly, a direct association between parental depression (T1) and child MVPA (T2) was not significant, *p* = 0.114.

To clarify which potential indirect associations linking parental depression (T1) and child BMI *z*-score were significant, the hypothesized unconstrained model was split into four mediation models (Table 2). Poor model-data fit was found for three nested models, assuming the following chains of associations: (1) parental depression (T1) → parental support provision (T1) → child MVPA (T2) → child BMI *z*-score (T2); (2) parental depression (T1) → child support receipt (T1) → child MVPA (T2) → child BMI *z*-score (T2); and (3) parental depression (T1) → child MVPA (T2) → child BMI *z*-score (T2). The poor fit obtained for the nested models (Table 2) suggests that these models should be rejected because the fit between the model and collected data is unacceptable; hence, it does not corroborate the assumed mediation chains.

The fourth nested model presented good model-data fit (Table 2) and did not differ from the hypothesized unconstrained model (ΔNFI = 0.004, ΔTLI = 0.004), therefore the fourth nested model may be accepted. The significant indirect effect (Table 2), included in this model, assumed three mediators operating sequentially. A higher level of parental depression (T1) was associated with a lower level of parental support provision (T1, the first mediator), which was associated with a lower level of child support receipt (T1, the second mediator), that in turn predicted a lower level of child MVPA (T2, the third mediator), which was associated with a higher level of child BMI *z*-score (T2; the dependent variable), the unstandardized indirect effect coefficient of 0.0001, *SE* < 0.0001, 99% CI (<0.0001, 0.0002). For the values of direct effects see Supplementary Table 1 and Figure 1.

Finally, the sensitivity analysis was conducted. The hypothesized unconstrained model was tested after removing all covariates from the model, except for T1-indicators of child MVPA and child BMI *z*-score. The analyses yielded a good fit and a similar pattern of direct and indirect associations as those obtained for the unconstrained model (Table 2).

## Discussion

This is the first study to describe the indirect across-individual prospective associations between parental depression and body mass of children aged 5–11 years old. Specifically, it was found that parents with more depressive symptoms were less likely to provide children with support for PA. In turn, their children perceived less support from their parents, and were less likely to be physically active, which in consequence predicted a higher level of child BMI *z*-score. The findings are congruent with the results of previous studies which indicated that parental depression is associated with a lower support provision and/or with lower child PA (Fernald et al., 2008; McConley et al., 2010; Wilson and Durbin, 2010).

The results of the present study are in line with social–ecological frameworks (Davison and Birch, 2001; Sallis et al., 2006), suggesting that health behavior change models should not only focus on the within-individual variables, but also on across-individual factors, referring to social support interactions. The across-individual (or dyadic) effects of social determinants of PA and its health outcomes have been thoroughly studied (Vilchinsky et al., 2011; Berli et al., 2016; Knoll et al., 2017). Although dyadic effects are suggested by the theoretical models (e.g., Davison and Birch, 2001), the across-individual approach is not so frequently accounted for in research investigating child PA and child body mass.

The majority of health behavior change models do not incorporate mental health indicators, such as depression. Yet, as highlighted in dual-process theory (Hagger, 2016), explicit (conscious) variables and implicit (automatic) affect-related factors should be considered when explaining behaviors. Similarly, the negative incidental affect concept (Rhodes and Kates, 2015) suggests that emotions (e.g., sadness or depression) can be predictors of health behaviors. Therefore, it is of high importance for research (and consequently, for practice) to account for mental health indicators when explaining health behaviors and their health-related outcomes, such as body mass. The health behavior taxonomy (Nudelman and Shiloh, 2015) suggests that psychological factors (such as positive and negative emotions) are crucial in clarifying why certain variables (e.g., social support) affect specific behaviors while others do not. Moreover, based on the health behavior taxonomy (Nudelman and Shiloh, 2015), it may be assumed that interventions addressing different psychosocial factors simultaneously (e.g., parental depression and support provision) may better explain changes in behaviors such as PA. Parents with high levels of depression symptoms should participate in treatment programs focusing not only on the symptoms, but also on enhancing parental practices (the National Research Council and Institute of Medicine, 2009). Such programs are likely to reduce adverse outcomes of parental depression in children (National Research Council and Institute of Medicine, 2009).

There are several limitations that need to be addressed when interpreting the results. First, the reliability of the applied MVPA measures was not high, yet similar to the reliability obtained in other studies (see Horodyska et al., 2018; Zarychta et al., 2019). Moreover, MVPA was measured with a self-report which may have limited validity due to the accuracy of the recall and social desirability effects (Andersen and Mayerl, 2017). Preferably, objective accelerometer-based measurement should be used (Dragsted et al., 2018); however, the feasibility of its use in large samples is limited. Second, the length of the time intervals between the baseline and follow-up measurements was relatively short. Moreover, the main limitation of the design refers to two measurement points only, instead of measuring the independent, the mediator, and the outcome variables at separate time points. For example, child MVPA and child BMI *z*-score were measured at T2. These two variables might have had an impact on each other (MVPA may determine BMI *z*-score and BMI *z*-score may determine the willingness to engage in MVPA). Similarly, the order in which parental support provision and child support receipt may operate cannot be established. Both variables were measured at the same time point. Therefore, future studies should use longer follow-up intervals and apply at least two follow-ups, with the independent variables, mediator variables, and dependent variables measured at different time points in order to establish a temporal precedence of variables. Next, the effect sizes obtained in this study were small, indicating weak associations between the predictor variables and the outcome variable. In consequence, the results should be interpreted with caution. Small effects on the main outcome variable could be expected, as the majority of variance of child BMI *z*-score at T2 is explained by the baseline BMI *z*-score. The gender homogeneity of the sample may affect the results since the majority of parental participants were mothers. The effects of parental gender have not been tested (due to the small number of father–child dyads). Further research should clarify whether gender differences exist. Any generalization to ethnically diverse populations should be made with caution as the analyzed sample was ethnically homogeneous (all participants were Caucasian). Ethnicity has been shown to predict positive outcomes in the context of caregiving (Parveen and Morrison, 2012) and it may moderate the associations between depression, social support, and health outcomes. Child illness is another potential moderator of support – health outcomes associations (Cipolletta et al., 2015); unfortunately, we did not account for other illnesses than those directly restricting child movement ability. Collected data did not allow to clarify if the recruited adults were biological parents, adoptive parents, or step-parents. Therefore, the role of heritability-related determinants, underlying the associations between depression and body mass, could not be controlled for. The findings have been obtained in the general population and should not be generalized to clinical populations. Future research should investigate if the patterns of dyadic associations are similar in clinical populations. Finally, several environmental factors which may influence child PA and body mass (e.g., availability of sweet and salty beverages, Luszczynska et al., 2013; accessibility to PA facilities, Horodyska et al., 2018) were not controlled in the study and should be included in future research.

## Conclusion

Concluding, it is important for health promotion practice to determine whether parental depressive symptoms may affect parents’ capabilities to provide support for PA to their children, and how the receipt of parental support explains child PA and child BMI *z*-score. Habitual behaviors, such as PA, may be difficult to change (Rothman et al., 2009). Therefore, the identification of modifiable determinants of PA is essential for the development of interventions improving childhood obesity rates. The results of this study suggest that parental depression may be the first determinant in the chain of parental support provision, support receipt, and child obesity-related behaviors (i.e., child MVPA). Thus, parental depression symptoms may be expected to (indirectly) explain child body mass. This pathway was confirmed in the present study, and may need to be addressed in health promotion programs aiming to reduce childhood obesity rates.

## Data Availability Statement

The datasets generated for this study are available on request to the corresponding author.

## Ethics Statement

This study was carried out in accordance with the recommendations of the Internal Review Board at the SWPS University of Social Sciences and Humanities, Wrocław, Poland. All participants gave written informed consent in accordance with the Declaration of Helsinki. The protocol was reviewed and approved by the Internal Review Board at the SWPS University of Social Sciences and Humanities.

## Author Contributions

KZ, AB, EK, MB, TR, and AL contributed to the study concept and design, and data acquisition. KZ and AL analyzed the data, interpreted the data, and drafted the manuscript. KZ, EK, MB, and AL wrote the sections of the manuscript. AB, TR, and CC revised the manuscript. All authors contributed to the manuscript revision, and read and approved the submitted version of the manuscript.

## Conflict of Interest

The authors declare that the research was conducted in the absence of any commercial or financial relationships that could be construed as a potential conflict of interest.
