# Supplementary material for: Parental Depression Predicts Child Body Mass via Parental Support Provision, Child Support Receipt, and Child Physical Activity: Findings From Parent/Caregiver–Child Dyads
Source: Front Psychol. 2020 Feb 7;11:161. doi: 10.3389/fpsyg.2020.00161 (PMC7019032; doi:10.3389/fpsyg.2020.00161)
Supplement: Supplementary file 1 [file Table_1.docx]

**Parental Depression Predicts Child Body Mass via Parental Support Provision, Child Support Receipt, and Child Physical Activity: Findings from Parent/Caregiver-Child Dyads**

**Supplement 1**

Supplement 1 reports direct effects for the hypothesized unconstrained model (Supplementary Table 1).

Supplementary Table 1

Standardized and unstandardized path coefficients and covariance coefficients for the hypothesized unconstrained model: the effects of parental depression, parental support provision, child support receipt, and child MVPA on child BMI *z*-score (*N* = 879 parent-child dyads).

| Path coefficients/covariance coefficients | Standardized estimate | Unstandardized estimate | *SE* | *p*-value |
| --- | --- | --- | --- | --- |
| **Predictors of study variables** | | | | |
| Depression (P, T1) 🡪 Support (P, T1) | - .121 | - 0.058 | 0.016 | < .001 |
| Depression (P, T1) 🡪 Support (Ch, T1) | - .010 | - 0.005 | 0.015 | .723 |
| Depression (P, T1) 🡪 MVPA (Ch, T2) | - .050 | - 0.177 | 0.112 | .114 |
| Support (P, T1) 🡪 MVPA (Ch, T2) | .120 | 0.893 | 0.277 | .001 |
| Support (Ch, T1) 🡪 MVPA (Ch, T2) | .082 | 0.545 | 0.254 | .032 |
| Support (P, T1) 🡪 Support (Ch, T1) | .543 | 0.608 | 0.032 | < .001 |
| MVPA (Ch, T1) 🡪 MVPA (Ch, T2) | .284 | 0.260 | 0.030 | < .001 |
| MVPA (Ch, T2) 🡪 BMI (Ch, T2) | - .035 | - 0.002 | 0.001 | .004 |
| BMI (Ch, T1) 🡪 BMI (Ch, T2) | .938 | 0.939 | 0.012 | < .001 |
| Depression (P, T1) 🡪 BMI (Ch, T2) | - .007 | - 0.001 | 0.002 | .572 |
| **Covariances** | | | | |
| MVPA (P, T1) 🡨🡪 MVPA (P, T2) | .615 | 200.200 | 12.880 | < .001 |
| MVPA (P, T1) 🡨🡪 MVPA (Ch, T1) | .178 | 92.872 | 17.550 | < .001 |
| MVPA (P, T2) 🡨🡪 MVPA (Ch, T1) | .063 | 28.906 | 14.915 | .053 |
| MVPA (P, T1) 🡨🡪 Gender (P, T1) | - .031 | - 0.219 | 0.242 | .365 |
| MVPA (P, T2) 🡨🡪 Gender (P, T1) | - .050 | - 0.316 | 0.212 | .136 |
| MVPA (Ch, T1) 🡨🡪 Gender (Ch, T1) | - .106 | - 1.446 | 0.450 | .001 |
| MVPA (Ch, T1) 🡨🡪 Gender (P, T1) | .031 | 0.319 | 0.339 | .347 |
| MVPA (P, T1) 🡨🡪 Depression (P, T1) | .058 | 7.874 | 4.553 | .084 |
| MVPA (Ch, T1) 🡨🡪 Depression (P, T1) | - .078 | - 14.879 | 6.425 | .021 |
| MVPA (P, T1) 🡨🡪 Age (P, T1) | - .013 | - 1.475 | 3.032 | .627 |
| MVPA (Ch, T1) 🡨🡪 Age (Ch, T1) | .061 | 2.210 | 1.198 | .065 |
| MVPA (Ch, T1) 🡨🡪 Age (P, T1) | .043 | 7.181 | 5.518 | .193 |
| Depression (P, T1) 🡨🡪 Gender (P, T1) | .030 | 0.079 | 0.088 | .372 |
| Depression (P, T1) 🡨🡪 Age (P, T1) | - .056 | - 2.376 | 1.415 | .093 |
| Age (Ch, T1) 🡨🡪 Age (P, T1) | .182 | 1.481 | 0.275 | < .001 |
| Gender (Ch, T1) 🡨🡪 BMI (Ch, T1) | - .038 | - 0.024 | 0.020 | .244 |
| Depression (P, T1) 🡨🡪 MVPA (P, T2) | .020 | 2.392 | 4.009 | .551 |
| Age (Ch, T1) 🡨🡪 BMI (Ch, T1) | .081 | 0.135 | 0.054 | .013 |
| Depression (P, T1) 🡨🡪 BMI (Ch, T1) | .002 | 0.013 | 0.291 | .963 |
| Depression (P, T1) 🡨🡪 BMI (P, T1) | .001 | 0.021 | 0.922 | .982 |
| MVPA (Ch, T1) 🡨🡪 BMI (Ch, T1) | - .003 | -0.085 | 1.116 | .939 |
| MVPA (P, T1) 🡨🡪 BMI (P, T1) | - .082 | - 6.107 | 2.478 | .014 |
| MVPA (P, T2) 🡨🡪 BMI (P, T1) | - .027 | - 1.777 | 2.163 | .411 |
| MVPA (Ch, T1) 🡨🡪 BMI (P, T1) | - .004 | - 0.438 | 3.549 | .902 |
| BMI (Ch, T1) 🡨🡪 BMI (P, T1) | .178 | 0.859 | 0.157 | < .001 |
| Gender (P, T1) 🡨🡪 BMI (P, T1) | - .265 | - 0.385 | 0.049 | < .001 |
| Age (P, T1) 🡨🡪 BMI (P, T1) | .144 | 3.399 | 0.752 | < .001 |

*Note.* T1 = Time 1, the baseline; T2 = Time 2, 7- to 8-month follow-up; P = Parent; Ch = Child; Support = social support provision (parents) and support receipt (children); MVPA = moderate-to-vigorous physical activity; BMI = body mass index *z*-score (children) and body mass index (parents).
